# The IsoArcH initiative: Working towards an open and collaborative isotope data culture in bioarchaeology

**DOI:** 10.1016/j.dib.2022.108595

**Published:** 2022-09-14

**Authors:** Esther Plomp, Chris Stantis, Hannah F. James, Christina Cheung, Christophe Snoeck, Lisette Kootker, Arwa Kharobi, Caroline Borges, Diana K. Moreiras Reynaga, Łukasz Pospieszny, Francesca Fulminante, Rhiannon Stevens, Aleksa K. Alaica, Adrien Becker, Xavier de Rochefort, Kevin Salesse

**Affiliations:** a Faculty of Applied Sciences, Delft University of Technology, Lorentzweg 1, 2628 CJ Delft, the Netherlands; b Department of Anthropology, National Museum of Natural History, Smithsonian Institution, Washington, DC, 20560, United States of America; c Analytical, Environmental & Geo-Chemistry, Department of Chemistry, Vrije Universiteit Brussel, Pleinlaan 2, 1050 Brussels, Belgium; d Maritime Culture Research Institute, Dept. of Art Sciences & Archaeology, Vrije Universiteit Brussel, Pleinlaan 2, 1050 Brussels, Belgium; e Geochemistry & Geology, Faculty of Science, Vrije Universiteit Amsterdam, De Boelelaan 1085, 1081 HV Amsterdam, the Netherlands; f CLUE+ Research Institute for Culture, History and Heritage, Vrije Universiteit Amsterdam, de Boelelaan 1105, 1081 HV Amsterdam, the Netherlands; g Faculty of Science and Technology, Department of Archaeology and Anthropology, Bournemouth University, Fern Barrow, Poole, Dorset BH12 5BB, United Kingdom; h Universidade Federal Rural de Pernambuco Recife, Pernambuco, Rua Dom Manuel de Medeiros, s/n - Dois Irmãos, Recife - PE, 52171-900, Brazil; i Department of Philosophy, The University of British Columbia, Vancouver, BC, V6T 1Z4, Canada; j Department of Anthropology, The University of British Columbia, Vancouver, BC, V6T 1Z4, Canada; k Institute of Archaeology and Ethnology, Polish Academy of Sciences, Rubież 46, 61-612 Poznań, Poland; l Department of Anthropology and Archaeology, University of Bristol, 43 Woodland Road, Bristol BS8 1UU, United Kingdom; m Institute of Archaeology, University College London, Gower Street, London WC1E 6BT, United Kingdom; n Division of Humanities, University Roma Tre, Piazza della Repubblica, 10, 00185, Roma, Italy; o UCL Institute of Archaeology, 31-34 Gordon Square, London WC1H 0PY, United Kingdom; p 3 Rue Bertrand de Montferrand, 33550 Langoiran, France; q xador.fr, Graulissac, 24130 Lunas, France; r Department of Anthropology, Faculty of Science, Masaryk University, Kotlářská 2, 611 37 Brno, Czech Republic

## 1 Introduction

From its inception in 2011, the IsoArcH initiative (https://isoarch.eu [1]) has been an altruistic effort to benefit and engage as many people as possible in the field of bioarchaeological science and beyond. The initiative quickly evolved from a small community to a multidimensional one of like-minded individuals promoting, in addition to their common scientific interests, best practices in data accessibility and ethics, collaborative knowledge, open research practices, reproducibility, transparency, scientific innovation, inclusion, and/or public awareness.

The cornerstone of the IsoArcH initiative is the IsoArcH database. The IsoArcH database is an isotope bioarchaeology database with samples (human, animal, plant materials) from all archaeological time periods and regions of the world. The isotopic data are complemented by detailed archaeological metadata, whenever available. Because of its collaborative nature and open access model, the IsoArcH database has brought together a variety of stakeholders interested in its services and results.

The IsoArcH initiative contribute to a more open and collaborative research culture in isotope bioarchaeology field. In this paper, we present the community structure of the IsoArcH initiative. We also reiterate the CARE (Collective benefit, Authority to control, Responsibility, and Ethics [2,3]) and FAIR (Findable, Accessible, Interoperable, Reusable [4,5]) principles and explain how they impact the IsoArcH community. Lastly, we argue that an open and collaborative culture within the scope of isotopic data in bioarchaeology is possible and that the IsoArcH initiative can help to move towards a more equitable and resilient isotope research culture in bioarchaeology.

## 2 The IsoArcH Community

**Fig. 1:**
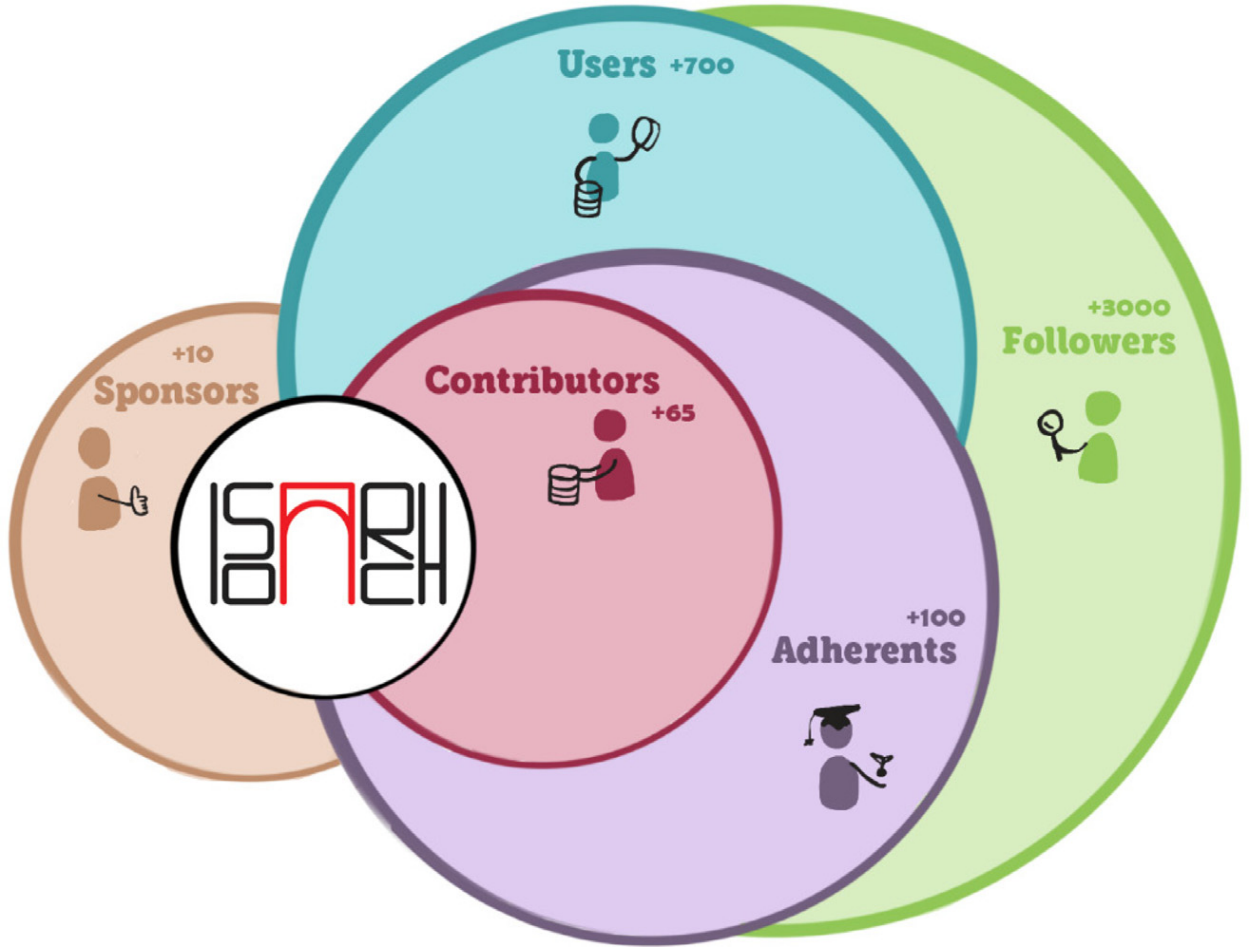
The five types of IsoArcH members with their estimated numbers indicated. In 2022, the community consists of over 3000 followers, more than 700 users, over 100 adherents, at least 65 contributors and more than 10 sponsors.

The IsoArcH community is composed of five different, but not mutually exclusive, types of engaged members: 1) adherents, 2) contributors, 3) users, 4) followers, and 5) sponsors (Fig. 1). Together, they constantly reshape the semantics and dynamics of the IsoArcH initiative.

### 2.1 Adherents

Adherents are experts in isotope bioarchaeology; they advocate for scientific cooperation and openness, knowledge sharing and transfer, as well as public outreach in their research areas (https://isoarch.eu/index.php/articles/). Through the IsoArcH association, adherents have access to virtual spaces for information and discussion. They can exchange experiences about current challenges, trending subjects, and controversial issues related to isotope bioarchaeology studies through different channels (including blogs and social media) and events (e.g., the “IsoArcH: Open Science in Archaeology” event [6]). Adherents collectively support the IsoArcH database, a common good for the entire field of bioarchaeological science and beyond. They contribute to the financial and scientific sustainability of the repository by collaboratively bearing the cost of website maintenance and services, securing the long-term growth of the repository. To date, over one hundred adherents are gathered under the umbrella of the IsoArcH association, consisting of academics and scholars from all career stages, public institutions, students, and enthusiasts from all over world. The association is open to all on request and after the payment of a membership fee (https://isoarch.eu/index.php/members/).

### 2.2 Contributors

Contributors are people who have shared research data with the IsoArcH database in the form of published (e.g., in scientific articles, book chapters, monographs) or released (e.g., in grey literature) isotope data, and their related (bio-)archaeological metadata. There are different profiles of contributors, with regular or occasional contributions. Contributors are appropriately credited for their work in all datasets. If contributors share work that they did not originally create, they must appropriately cite and credit the original work from which the data originate and ensure that they comply with the licensing terms of the original work. Upon request, contributors can receive a Digital Object Identifier (DOI) for the datasets they submit. DOIs allow datasets to be efficiently referenced, shared, and cited, facilitating long-term access of data, and increasing their findability and discoverability. DOIs are generated by DataCite Fabrica (https://doi.datacite.org/), whose access is contractually granted to the IsoArcH association by INIST-CNRS (https://www.inist.fr/), a member of the DataCite consortium (https://datacite.org/). They resolve to landing pages on the IsoArcH website (https://doi.isoarch.eu/), allowing to get all information necessary for identifying and citing the datasets (e.g., contributor names, abstracts, keywords, full bibliographic citations, DOI displayed as URL, data, etc.), thus providing recognition for the effort made by contributors to share data with the IsoArcH database.

### 2.3 Users

Users represent a very diverse group, both in terms of background and expectations. The bulk of the users includes undergraduates, postgraduates, academics, and scholars. Users can consult and query the IsoArcH database, as well as export the desired data without technical or financial restrictions. Users also contribute by revealing possible computer bugs, errors in (meta)data entry, and typos in the textual content of the website or database. Users seek information and guidance on the services provided by the IsoArcH initiative and database, wanting to understand the advantages of the IsoArcH database over competing solutions. Most users connect with the IsoArcH team by email (contact@isoarch.eu). The number of registered users currently exceeds 700.

### 2.4 Followers

Followers form a group of over 3000 people on Twitter (https://twitter.com/isoarch_eu) and Facebook (https://www.facebook.com/isoarch). They have diverse interests, beyond isotope bioarchaeology. Followers share, support, and/or are concerned with the ideas and ideals promoted by the IsoArcH initiative. Through these social networks, followers stay tuned and have access to the latest updates and news about the development of the database, the activities of the association, and the events of the initiative. Followers provide encouragement in many positive and vibrant ways, giving strength to the teams involved in the development of the IsoArcH database or in the running of the IsoArcH association. Their timely interventions on social networks are also a driving force for the development of new action plans within the IsoArcH initiative.

### 2.5 Sponsors

Sponsors and benefactors are key partners of the IsoArcH community. Together with the adherents, sponsors provide funds used to improve IsoArcH's web applications and infrastructure and offer visibility and promotion of the IsoArcH database. Furthermore, sponsors give benefits for the IsoArcH adherents. One example is the agreement between the IsoArcH association and Elsevier's Data in Brief journal (https://www.journals.elsevier.com/data-in-brief). All adherents can receive a full waiver of the article publication charge (APC) for publishing gold open access data articles in Data in Brief. This agreement is valid from 2022 until the end of 2025. In conjunction with the establishment of this agreement, the IsoArcH database has been identified as a scientific data repository endorsed by Elsevier (https://fairsharing.org/graph/3402).

## 3 Principles of IsoArcH

### 3.1 CARE

The CARE (Collective benefit, Authority to control, Responsibility, and Ethics) principles are designed to centre data governance for Indigenous communities, and address issues that are relevant to many communities and populations involved in knowledge creation and decision-making processes. These principles are people- and purpose-oriented. They require engagement with communities and populations, where possible, to address the cultural, ethical, legal, and social dimensions associated with data collection and reuse [2,3]. They also emphasize the need for inclusive data governance as data play a critical role in fostering innovation, knowledge creation, decision-making processes, and self-determination of people [2,^3^,^7^,8]. In addition, the CARE principles offer guidance for achieving socially meaningful outcomes by recognizing that the epistemic context of acquired data can negatively influence data ecosystems, institutions, people, and individuals [7], [8], [9].

The IsoArcH initiative supports the CARE principles and their supporting concepts. Although it does not create data per se, the IsoArcH database stores a wide range of sensitive data, including biological information of archaeological human remains, which must be handled with the utmost dignity and care. To achieve equitable research outcomes, it is important that data submitted to the IsoArcH database come from research works compliant with local governance processes, and that the creation and reuse of data reflect people's values (i.e., the “Collective benefit” of the CARE principles). People and stakeholders involved in the research study must have control over the data processes, allowing them to actively participate in data stewardship. When data provenance or data sharing permissions are unclear, the submission to the IsoArcH database may be put on hold until clarification is provided, or even denied (i.e., the “Authority to control” of the CARE principles). The isotope bioarchaeology community has a responsibility to nurture relationships with communities and populations from whom the data originates and must maximize benefits while minimising harm and promoting justice (i.e., the “Responsibility” of the CARE principles). Ethics in data science are essential and the IsoArcH community is committed to developing measures to ensure that all data entered into the online database is collected in an ethical manner (i.e., the "Ethics" of the CARE principles).

### 3.2 FAIR

The FAIR (Findable, Accessible, Interoperable, Reusable) principles optimize the use of existing research data [4,5]. They recommend that data include sufficiently rich metadata and a unique, persistent identifier. They advise that metadata and data be understandable to both humans and machines, and that data are stored in a data repository. Access to data should be allowed under well-defined preconditions, including any relevant restrictions on data related to humans or communities (see CARE principles). Community accepted languages, formats, and vocabularies should be used in metadata. In this regard, metadata standardization is essential to achieve a common understanding of the meaning and structuring of data. Formats in which data are shared should preferably be open to increase the longevity of the datasets and to prevent the data from being locked into a proprietary system, so users may be able to use, modify, and distribute the materials for any purpose. The principles also request that data and data collections be appropriately licensed and accompanied by accurate documentation of their provenance. In other words, the FAIR principles provide an effective framework for fostering and developing research data services such as the IsoArcH database (see Table 1 for more details about these principles).

**Table 1:** The FAIR principles with a short explanation on how to achieve them.

Findable	For datasets to be “Findable” they must be accompanied with a persistent identifier (such as a DOI) and sufficient metadata (such as creator, date, methods, standards, accuracy, and precision).
Accessible	The “Accessible” in FAIR is not the same as “open”, as FAIR only requires having adequate procedures in place for others to request access to data archived on a data repository such as IsoArcH; it does not require this access to be openly granted. In circumstances where data cannot be publicly shared (see the CARE principles), researchers can still make the information about the dataset (metadata) publicly available and keep the data under restricted access. The statement “Data available upon request” in articles does not follow the FAIR principles, as there is no persistent identifier associated with the data, and access procedures are unclear and changeable, with increasing difficulties in contacting those responsible for these requests.
Interoperable	Data that are “Interoperable” follow a common format and consistent vocabulary, such as metadata standards (a common understanding of the meaning or structuring of the data). Therefore, interoperable data is easier to understand and reuse. The format in which the data is shared is preferably open: relying on proprietary formats can make the data inaccessible to those without access to licences (e.g., Microsoft Word). Open formats, such as .txt, .md or .pdf are also likely to have increased longevity (with some of these formats being more machine interoperable than others).
Reusable	Data are “Reusable” when accompanied by documentation and a licence. Documentation can range from a short README file to more extensive documentation about the data, such as laboratory notes and full protocols. A licence indicates the terms and conditions regarding sharing and re-use of data and software. Creative Commons licences (https://creativecommons.org/licenses/) are generally used to license data.

The IsoArcH database promotes the reuse of research data according to the FAIR principles. The IsoArcH database can assign permanent identifiers such as DOIs to datasets. Datasets with DOIs are linked to landing pages on the IsoArcH website containing basic metadata, such as keywords, time period(s), geographic location(s), and references to other search results (“Findable” and “Accessible” in FAIR principles). Landing pages may also store documentation about the datasets. The IsoArcH database can process files with data from open-source office suites, such as Open Office (“Interoperable” in FAIR principles). The IsoArcH database and its contents are distributed under the terms of a copyleft open-source licence, i.e., the BY-NC-SA 4.0 International Creative Commons licence (“Reusable” in FAIR principle). Lastly, the IsoArcH database is listed as a repository on FAIRsharing (https://fairsharing.org/3319), a recognized resource for data standards, databases, and policies.

In order to be adequately combined into composite datasets, the data must be standardized [10], [11], [12], [13], [14], [15], [16]. This requires access to transparent and detailed documentation on sample selection, chemical treatments, measurement procedures, standards, and calibrations/calculations used, blank and interference corrections, accuracy and precision, and so forth [10], [11], [12],^14^,[17], [18], [19], [20], [21], [22], [23], [24], [25], [26], [27], [28], [29], [30]. Adopting a common terminology will improve interoperability and reusability of data [10,31]. The IsoArcH initiative provides a means to address these standardization issues, which can only be achieved with input from the broader isotope bioarchaeology community.

Developments are underway to make the IsoArcH database an open-source database. IsoArcH database applications with codebases will be free to view, download, modify, distribute, and reuse. The use of open-source software packages for the new upcoming version of the database will facilitate this release. This transformation will increase the interoperability and reusability of data, ease the contributions of community members, and make the database more sustainable in the long term.

### 3.3 Openness

The IsoArcH initiative facilitates openness in the isotope bioarchaeology community by providing open access to data submitted to the database. IsoArcH recognizes data as a valuable research output and promotes data preservation and sustainability, visible data citations, and increased impact of data-related works. The IsoArcH initiative responds to UNESCO's recommendations on open science, which state that equity is key to ensure “fair and reciprocal sharing of scientific inputs and outputs and equal access to scientific knowledge to both producers and consumers of knowledge” [32]. Sharing research outputs through more sustainable and open venues is increasingly important to ensure that research participation is not restricted based on personal or institutional resources.

The IsoArcH association also provides its members with options to release their data in data articles published by open access journals. A notable example is the special issue “IsoArcH best practices for managing and sharing data” published in Elsevier's Data in Brief journal (https://www.sciencedirect.com/journal/data-in-brief/special-issue/100GHPQPRDW). Another example is the current and forthcoming agreements with scientific journals allowing IsoArcH adherents to publish open access data articles for free. This ensures, among other things, that members of less-funded research institutes or groups, especially those from low- and middle-income countries, are not excluded from the sharing process.

The IsoArcH database helps researchers to comply with recent changes in research evaluation that focus on whether research results have been made openly available instead of focusing on where they were published. Open access policies incorporating these evaluation recommendations are increasingly common in scientific communities, research centres, and research funding agencies. Some examples include the San Francisco Declaration of Research Assessment (https://sfdora.org/), Plan S (https://www.coalition-s.org/), European Commission (https://ec.europa.eu/), National Institutes of Health (https://grants.nih.gov/), Dutch Research Council (https://www.nwo.nl/en/), Polish National Science Centre (https://www.ncn.gov.pl/en/), and UK Research and Innovation (https://www.ukri.org).

## 4 Call to Action

We, the IsoArcH community, as part of the broader isotope bioarchaeology community, are committed to an open and collaborative culture of isotope data that will promote better access to data and diversity in scientific research. We trust that this change in our approach to data curation will have a positive effect on the pace and direction of scientific progress in bioarchaeological science and beyond. We invite all members of the isotope bioarchaeology community to join us, so that we all contribute to a more accessible, inclusive, and participatory practice of science. The IsoArcH initiative will continue to facilitate further discussions by organising several sessions for the isotope bioarchaeology community in the upcoming years. It also welcomes feedback (contact@isoarch.eu) on the ongoing technical developments that will improve the data collection and curation processes.

## 5 Conclusion

The isotope bioarchaeology community is on a long journey towards more open and equitable access to isotope data. Initiatives such as IsoArcH are critical to provide the technical infrastructure and collaborative research network necessary to achieve these goals. By implementing the CARE principles, the IsoArcH initiative is committed to acknowledge the people behind the data; whether it be those from whom they originated, those who deposit them, or those who find a use for them. Following the FAIR principles, the IsoArcH initiative contributes to building the future of archaeological research from mining its past, stressing the need for data sustainability and reusability. The implementation of both CARE and FAIR principles is important to our field as bioarchaeological samples are non-renewable and isotope analyses are destructive. It is of utmost importance to maximize their value and optimize their use by the wider research community. The IsoArcH initiative aims to support research involving isotope bioarchaeology, and to create synergy for intra- and inter-disciplinary collaboration between various research groups. We invite members of the isotope bioarchaeology community to participate in discussions intended to enhance the impact of isotope data. By uniting our efforts, the road may feel less steep and more worthwhile.

## CRediT authorship contribution statement

**Esther Plomp:** Conceptualization, Writing – original draft, Writing – review & editing, Project administration. **Chris Stantis:** Writing – original draft, Writing – review & editing, Project administration. **Hannah F. James:** Writing – original draft, Writing – review & editing, Project administration. **Christina Cheung:** Writing – original draft, Writing – review & editing, Project administration. **Christophe Snoeck:** Writing – review & editing, Project administration. **Lisette Kootker:** Writing – review & editing, Project administration. **Arwa Kharobi:** Writing – review & editing, Project administration. **Caroline Borges:** Writing – review & editing, Project administration. **Diana K. Moreiras Reynaga:** Writing – review & editing, Project administration. **Łukasz Pospieszny:** Writing – review & editing, Project administration. **Francesca Fulminante:** Writing – review & editing, Project administration. **Rhiannon Stevens:** Writing – review & editing, Project administration. **Aleksa K. Alaica:** Writing – review & editing, Project administration. **Adrien Becker:** Software, Data curation, Project administration. **Xavier de Rochefort:** Software, Data curation, Project administration. **Kevin Salesse:** Conceptualization, Writing – original draft, Writing – review & editing, Software, Data curation, Project administration.

## Declaration of Competing Interest

The authors declare that they have no known competing financial interests or personal relationships that could have appeared to influence the work reported in this paper.
